# Assessing Antenatal Care and Newborn Survival in Sub-Saharan Africa within the Context of Renewed Commitments to Save Newborn Lives

**DOI:** 10.3934/publichealth.2016.3.432

**Published:** 2016-06-24

**Authors:** Henry V. Doctor

**Affiliations:** World Health Organization, Regional Office for the Eastern Mediterranean, Cairo, 11371, Egypt

**Keywords:** antenatal care, neonatal mortality, newborn care, quality care, sub-Saharan Africa

## Abstract

Antenatal care (ANC) is one of the key interventions of the *Every Newborn* action plan to improve newborn health and prevent stillbirths by 2035. However, little is known about its relationship with neonatal mortality in sub-Saharan Africa since the 1990s. We use data from 54 Demographic and Health Survey (DHS) from 27 countries to make comparisons of neonatal mortality by ANC attendance. Each country had two surveys that were categorized as ‘earliest surveys’ (i.e. conducted since 1990 but before 2010) and ‘latest surveys’ (from 2010 to 2014). Multi-level logistic regression model and meta-analysis were applied on 1.1 million births that occurred among women in the 5 years preceding the surveys. Overall neonatal mortality rate (NMR) was 37.7 (95% CI, 37.4–38.1) deaths per 1000 live births; NMR in the earliest surveys were 46.0 (95% CI, 45.4–46.7) and 33.4 (95% CI, 33.0–33.8) deaths per 1000 live births in the latest surveys. The overall NMR was also 10% higher than expected NMR (37.7 vs 34.3 deaths per 1000 live births). NMR was 2.2 times higher among births of women with no ANC compared to those who had at least one ANC visit (42.5 vs 19.6 per 1000 live births). After adjusting for place of delivery, maternal age at birth, relative household wealth, residence, mother's education, marital status, birth order, sex of child, and period of survey, the overall odds ratio (OR) demonstrated that women with at least one ANC visit were 48% less likely to report neonatal deaths (OR: 0.52; 95% CI: 0.47–0.57) than women who did not receive ANC. NMR was 27% less likely to occur during the latest surveys than during the earliest surveys (OR: 0.73; 95% CI: 0.71–0.75). We discuss these results within the context of calls for continued efforts to deploy interventions aimed at improving the quality of maternal and newborn care.

## 1. Introduction

Antenatal care (ANC) has been recommended for a considerable time to be effective in improving maternal and newborn survival [1]; and is one of the key interventions of the *Every Newborn* action plan to improve newborn health and prevent stillbirths by 2035 [2]. Interventions received during ANC visits such as iron folic acid supplementation, tetanus toxoid injections, other diagnostic tests as well as pregnancy and newborn counseling have been recommended and accepted as key interventions to improve maternal and child health [1],[3]. To ensure improved maternal and newborn health, the World Health Organization recommends that women should attend at least four focused ANC visits. The first visit should preferably take place during the first trimester [4]. However, in many developing countries, few women attend at least four ANC visits.

While the association between ANC and improved maternal and child health outcomes has been recorded in a number of countries (e.g. [3],[5]), ANC still remains out of reach for majority of women particularly in developing countries. In particular, lack of ANC attendance ultimately leads to limited utilization of skilled attendance at delivery. Several factors account for this limited utilization in health services such as ANC and skilled attendance at delivery. Socio-cultural factors, perceived benefit or need of health services, economic accessibility, and physical accessibility are some of the key challenges experienced by women in seeking health care [6]. For example, some cultural beliefs encourage women to deliver alone as a sign of “strength” and discourage ANC visits or skilled attendance at delivery [7]. Other women who would like to attend ANC may not be able to do so due to long distances from their homes to facilities or the inability to pay for health care costs. Other women may find some health staff to be unfriendly such as the case in northern Nigeria where women in one community shunned visiting clinics because nurses at a General Hospital ignored them and called them “villagers who don't know anything” [8].

In order to improve maternal and newborn survival, a number of countries in sub-Saharan Africa have committed to deploying interventions that encourage women to attend ANC and utilize health services. This became increasingly important within the context of the health-related Millennium Development Goals (MDG); and the fact that newborn health and still births are part of the “unfinished agenda” of the MDGs for women's and children's health [2]. Newborn deaths still account for close to half of the global under-5 deaths [9] and newborn mortality and stillbirths have a great visibility in the health-related Sustainable Development Goals.

Against this background, the main objective of this paper is to assess the neonatal mortality differences across countries with a particular focus on disparities between women who attended at least one ANC and those who did not receive ANC. To do so, we expand the results from existing literature which focus on selected countries by combining all data available from the early 1990s and those available since 2010 from the Demographic and Health Surveys (DHS) into a pooled data set covering 1.1 million births under the age of 5 across 54 surveys in 27 sub-Saharan African countries. This approach provides a comparison of the trends in neonatal mortality by whether women received ANC by comparing results from surveys conducted during the onset of the MDGs in the 1990s and surveys conducted toward the end of the MDGs in 2015.

## 2. Data and Methods

### 2.1. Data

This paper uses publicly de-identified individual-level data from the DHS (http://www.dhsprogram.com/), nationally representative population-based surveys with a historical focus on fertility and reproductive health but also cover a variety of child health outcomes and household characteristics beyond child survival outcomes [10]. The DHS Program is largely funded by the U.S. Agency for International Development and the data offer various indicators including neonatal mortality and household relative wealth calculated using information on households' assets and other possessions. By September 2015, more than 330 surveys had been conducted in developing countries, of which 133 surveys were conducted in sub-Saharan Africa. Of the 133 sub-Saharan African surveys, 10 had a single survey. We finally used the 27 sub-Saharan African countries that had the earliest and most recent surveys (n = 54) with neonatal mortality estimates provided by ANC attendance (ANC). The “earliest surveys” are defined as those conducted from 1990 onwards but before 2010; and “most recent” surveys (or “latest” surveys) are those conducted from 2010 to 2014, close to the MDG deadline of 2015. By implication, countries that had only one DHS during this period were not included in the analysis.

The time interval between the earliest and most recent DHS data provides sufficient time to observe reasonable changes in neonatal mortality. Time intervals between the earliest and latest surveys ranged from 5 to 23 years, averaging 13.7 years of observation time (Table 1).

### 2.2. Methods

#### 2.2.1. Variables

The outcome variable, neonatal mortality, is the probability of dying within the first 28 days of birth, and expressed per 1000 live births. The information on age at death for the live births that took place within the 5 years preceding the DHS was utilized to identify neonatal deaths. Neonatal mortality was coded as ‘1’ for neonates who died before 28 days and ‘0’ for those who survived the 28 days. The key independent variable is whether the woman received at least one ANC; coded as 1 if “yes” and “0” otherwise. This dichotomy was used due to low percentages of women who attended least four ANC visits. Other independent variables include relative household wealth, a proxy for socio-economic status (SES) and expressed as wealth quintiles. The wealth quintile is calculated using principal components analysis (PCA) based on data from the household's ownership of consumer goods; dwelling characteristics; type of drinking water source; toilet facilities; and other characteristics that are related to a household's SES. The PCA method has been used extensively in less developed countries [11],[12].

**Table 1.** Countries and Demographic and Health Surveys used in the analysis for 27 sub-Saharan African countries.

Country	Earliest Survey		Most recent Survey		Observation years
Western Africa (n = 12)					
Benin	1996		2011–12		16
Burkina Faso	1993		2010		17
Cote d'Ivoire	1994		2011–12		18
Ghana	1993		2014		21
Guinea	1999		2012		13
Liberia	2007		2013		6
Mali	1995–96		2012–13		17
Niger	1998		2012		14
Nigeria	1990		2013		23
Senegal	1997		2014		17
Sierra Leone	2008		2013		5
Togo	1998		2013–14		16
Middle Africa (n = 3)					
Cameroon	1991		2011		20
Congo (Brazzaville)	2005		2011–12		7
Congo Democratic Republic	2007		2013–14		7
Eastern Africa (n = 10)					
Comoros	1996		2012		16
Ethiopia	2000		2011		11
Kenya	1993		2014		21
Malawi	1992		2010		18
Mozambique	1997		2011		14
Rwanda	1992		2010		18
Tanzania	1996		2010		14
Uganda	1995		2011		16
Zambia	1996		2013–14		18
Zimbabwe	1994		2010–11		17
Southern Africa (n = 2)					
Lesotho	2004		2009		5
Namibia	1992		2013		21
			
**Summary statistics**					
Minimum observation time (years)	5				
Maximum observation time (years)	23				
Mean observation time (years)	13.7				
Standard deviation	5.1				

Note: Latest surveys defined as those from 2010 with the exception of Lesotho (2009) which is included to increase the number of surveys in Southern Africa.

Source: The DHS Program; Last accessed 4 February 2016.

#### 2.2.2. Statistical analysis

The most recent DHS data from the 27 countries were pooled into a data set covering 704,441 births occurring in the 5 years preceding the surveys. Similarly, the pooled earliest data set covered 378,966 births; yielding a total of 1.1 million births. The pooled data set utilized birth history files where each woman was asked for the date of birth (month and year) of each live-born child, the child's sex, whether the child was still alive (and if the child had died) the age at death (in days for the first month, in months if the deaths occurred between 1 and 24 months, and in years thereafter). These data allowed child deaths to be located by time and by age [10]. We used multilevel logistic regression model using Stata (version 14, StataCorp LP, College Station, TX, USA) to estimate the magnitude of association in form of odds ratios (ORs) between neonatal mortality and ANC, adjusting for residence (urban/rural); women characteristics (education, marital status, place of delivery, age at birth); wealth status; survey indicator; and child-specific characteristics (birth order, sex). In particular, multilevel models were constructed using the mixed effects modeling procedure where data have been collected in nested units. Sampling cluster was included in the model as nested random effects with country modeled as fixed effects.

The adjusted effects of ANC on neonatal mortality from the multilevel logistic regression model for each country were used to conduct meta-analysis in Stata to develop a forest plot of the adjusted pooled effect (i.e. women with at least one ANC compared to women with no ANC) on NMR across 27 countries. The pooled ORs with associated 95% confidence intervals (CI) were estimated using Mantel-Haenszel statistical methods. Heterogeneity among the surveys was assessed using *I^2^* statistics, a measure of the proportion of total variability explained by heterogeneity rather than chance expressed as a percentage [13]. Roughly, an *I^2^* of 0–40% represents no or little heterogeneity, 30–60% moderate heterogeneity, 50–90% substantial heterogeneity, and 75–100% considerable heterogeneity [14]. The meta-analysis applied random effects analytical model due to the moderate heterogeneity (> 55%) among the survey results. Observed NMRs were compared with expected NMRs which were obtained after adjusting for the risk factors in the regression model.

## 3. Results

Table 2 presents frequency of ANC and other birth-related characteristics for births occurring 5 years before the surveys. About 21% of births occurred among mothers who had at least one ANC visit. About 22% of the births occurred in a health facility and close to a quarter of births occurred to mothers aged under 20 years. About one in four births (23.0%) occurred among women in the lowest quintile of the wealth index; and 74.5% occurred in the rural areas. There were almost equal numbers of births occurring among women with no formal schooling and those with at least primary schooling. Nine out of 10 women were married or living with a partner and about 40% were births of order 2–3. The ratio of male to female births was 1.02, consistent with sex ratios reported earlier from African population [15]; and 35% of all births occurred during the earliest surveys.

**Table 2.** Prevalence of antenatal care and other birth-related characteristics of most recent births for 27 sub-Saharan African countries.

Characteristics	N (weighted) (%)
Pregnancy characteristics	
Antenatal care	
None	857,493 (79.2)
At least once	225,554 (20.8)
Place of delivery	
Home	850,894 (78.5)
Health facility	232,513 (21.5)
Mother's age at birth of baby	
< 20	260,347 (24.0)
20–24	342,073 (31.6)
25–29	249,173 (23.0)
30+	231,792 (21.4)
Household characteristics	
Wealth quintile	
Lowest	239,289 (23.0)
Second	232,064 (22.3)
Third	216,931 (20.8)
Fourth	198,203 (19.0)
Highest	154,887 (14.9)
Residence	
Rural	807,031 (74.5)
Urban	276,376 (25.5)
Woman characteristics	
Mother's education	
None	547,172 (50.5)
At least primary	536,111 (49.5)
Married/Living together	
No	103,738 (9.6)
Yes	979,637 (90.4)
Child-specific characteristics	
Birth order	
1	289,737 (26.7)
2–3	401,468 (37.1)
4+	392,203 (36.2)
Child's sex	
Male	548,244 (50.6)
Female	535,163 (49.4)
Survey characteristics	
Round of survey period	
Earliest	378,966 (35.0)
Latest	704,441 (65.0)
	
**All births**	**1,083,408 (100.0)**

Note: Numbers between categories vary due to missing values.

Table 3 presents neonatal mortality rates (NMR) for the earliest and most recent surveys and by ANC attendance. Results show that by the most recent surveys, NMR had declined from the level observed during the earliest surveys. NMR was 2.2 times higher among births of women with no ANC than among births of women who had ANC (42.5 vs 19.6 per 1000 live births). The overall NMR was 37.7 (95% CI, 37.4–38.1) deaths per 1000 live births.

**Table 3.** Neonatal mortality rates with 95% CI by survey period and antenatal care attendance.

Characteristics	Neonatal mortality rate (deaths / 1000 live births)
Survey period	
Earliest survey	46.0 (45.4–46.7)
Most recent survey	33.4 (33.0–33.8)
Antenatal care attendance	
None	42.5 (42.1–42.9)
At least once	19.6 (19.0–20.1)
**All births**	**37.7 (37.4, 38.1)**

Country variations in NMRs by ANC attendance and the number of births included in the analysis are presented in Table 4. The lowest NMR among births of women with no ANC was in Namibia (25.5 deaths per 1000 live births); and the highest in Mali (63.9 deaths per 1000 live births). Among women with at least one ANC visit, the lowest NMR was in Kenya at 13.7 deaths per 1000 live births and the highest in Sierra Leone at 27.7 deaths per 1000 live births. Among all births and irrespective of whether women attended ANC or not, NMR was lowest in Namibia at 23.3 deaths per 1000 live births and highest in Mali at 59.4 deaths per 1000 live births. The difference between the country with the lowest NMR and the country with the highest NMRs among births of women with no ANC was 38.4 deaths per 1000 live births. Similarly, the difference was 14.0 deaths per 1000 live births among births of women with ANC. The overall difference for the country with the lowest and highest NMRs was 36.1 deaths per 1000 live births. The ratio of NMR among births of women with no ANC to those with at least one ANC visit ranged from 1.4 in Namibia to 3.3 in Niger. Ten countries had this ratio above the average (2.2) for all the countries whereas the remaining 17 countries had their ratio below the average.

**Table 4.** Neonatal mortality rates among births of women by antenatal care attendance for 27 countries, 1990–2014.

Country	NMR among births of women with no ANC		NMR among births of women with at least one ANC visit	NMR among births of all women		Ratio of NMR by ANC attendance	Number of all births
(1)	(2)		(3)	(4)		(5) = (2)/(3)	(6)
Niger	49.7		15.1	45.5		3.3	48,840
Burkina	50.8		16.3	44.1		3.1	50,211
Liberia	43.9		15.1	36.3		2.9	40,860
Rwanda	46.1		16.8	39.8		2.7	33,952
Ethiopia	57.6		22.2	55.4		2.6	57,918
Malawi	40.6		15.4	34.4		2.6	65,849
Ghana	37.4		15.6	31.1		2.4	18,945
Mali	63.9		26.9	59.4		2.4	47,256
Mozambique	49.9		20.4	44.4		2.4	40,559
Cote d I'voire	48.2		20.6	43.3		2.3	28,583
Benin	30.2		14.6	27.0		2.1	39,659
Cameroon	37.8		17.9	33.1		2.1	32,344
Guinea	54.6		26.2	48.3		2.1	29,609
Uganda	35.7		17.1	32.2		2.1	38,713
Congo Brazzaville	28.6		14.6	24.5		2.0	26,718
Kenya	27.9		13.7	24.2		2.0	56,281
Congo Dem. Republic	34.2		17.7	31.0		1.9	57,863
Nigeria	48.8		25.1	45.1		1.9	84,918
Sierra Leone	53.4		27.7	47.1		1.9	65,159
Senegal	38.9		21.5	34.9		1.8	43,043
Togo	39.2		21.4	35.6		1.8	30,317
Lesotho	45.6		27.5	39.8		1.7	14,271
Zambia	31.0		18.0	27.3		1.7	46,926
Zimbabwe	26.4		15.6	23.6		1.7	20,513
Comoros	32.9		21.9	31.0		1.5	11,986
Tanzania	33.1		22.8	30.2		1.5	33,981
Namibia	25.5		17.6	23.3		1.4	18,134
**All countries**	**42.5**		**19.6**	**37.7**		**2.2**	**1,083,408**

Neonatal mortality rate for all the 27 countries was 10% higher than expected NMR (37.7 vs 34.3 deaths per 1000 live births) (Table 5). In 21 out of 27 countries (or 77.8%), NMR was higher than expected; ranging from 1% in Mali to 16% in Comoros and Mozambique. In 3 countries (Guinea, Cote d'Ivoire, and Nigeria), there were no differences between observed and expected NMRs (Relative Risk = 1.00). In 3 countries (Ghana, Liberia, and Malawi), NMR was lower than expected.

Multi-level logistic regression results are presented in Table 6. Results adjusted for selected variables show that newborns of mothers with at least one ANC visit were less likely to die during the neonatal period than newborns of mothers with no ANC [odds ratio (OR): 0.53; 95% confidence interval (CI): 0.50–0.56]. Newborns of mothers aged 20–24 years were 35% less likely to die (OR: 0.65; 95% CI: 0.63–0.67) than newborns of mothers aged under 20 years. Similarly, newborns of mothers aged 25–29 years and 30 years and above were 47% and 43%, respectively, less likely to die than newborns of mothers aged under 20 years. Newborns in urban areas were 14% less likely (OR: 0.86; 95% CI: 0.84–0.89) to die than newborns in rural areas. Mothers with at least primary schooling and those who were married were less likely to report neonatal deaths (OR = 0.81 and OR = 0.91, respectively). Newborns with birth order of 2–3 and 4+ were 28% (OR: 0.72; 95% CI: 0.70–0.74) and 10% (OR: 0.90; 95% CI: 0.87–0.94), respectively, less likely to die than newborns of birth order 1. Female newborns were 27% (OR: 0.73; 95% CI: 0.71–0.74) less likely to die than male newborns. During the most recent surveys, the odds of newborns dying were lower than during the earliest surveys (OR: 0.73; 95% CI: 0.71–0.75). However, the reduction in the odds of dying over time for newborns of mothers who had at least one ANC compared to newborns of mothers with ANC was not statistically significant, OR: 0.97 (95% CI: 0.90–1.04).

**Figure 1.**
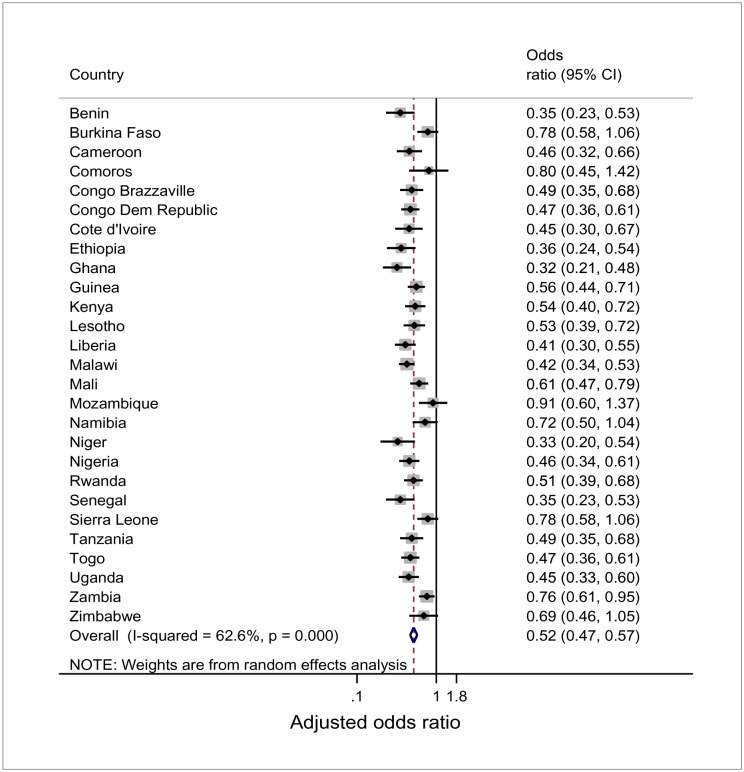
Forest plot of adjusted odds ratios of neonatal mortality of births of mothers who had at least one ANC compared to mothers who had no ANC for 27 countries.

The overall meta-analysis (Figure 1) of NMRs among newborns of mothers who had at least one ANC visit compared to mothers with no ANC includes 1.1 million births for 27 countries. That is, Figure 1 displays the pooled ORs from multi-level logistic regression analyses for each country, similar to results presented in Table 5. The pooled OR demonstrated that neonates of mothers who had at least one ANC were 48% less likely to die than neonates of mothers who had no ANC (OR: 0.52, 95% CI: 0.47–0.57). The results showed moderate heterogeneity between the surveys (*I^2^* = 62.6%).

**Table 5:** Observed and expected neonatal mortality rate with relative risks for 27 countries, 1990–2014.

Country		Neonatal mortality rate		Relative Risk
		Observed	Expected	
Comoros		31.0	26.7	1.16
Mozambique		44.4	38.3	1.16
Burkina Faso		44.1	40.3	1.09
Benin		27.0	24.7	1.09
Niger		45.5	42.5	1.07
Kenya		24.2	22.7	1.07
Cameroon		33.1	31.2	1.06
Zambia		27.3	26.1	1.05
Namibia		23.3	22.3	1.04
Lesotho		39.8	38.4	1.04
Zimbabwe		23.6	22.8	1.04
Sierra Leone		47.1	46.0	1.02
Senegal		34.9	34.1	1.02
Congo Dem. Republic		31.0	30.3	1.02
Ethiopia		55.4	54.2	1.02
Uganda		32.2	31.6	1.02
Togo		35.6	35.0	1.02
Congo Brazzaville		24.5	24.1	1.02
Rwanda		39.8	39.4	1.01
Tanzania		30.2	30.0	1.01
Mali		59.4	59.1	1.01
Cote d'Ivoire		43.3	43.2	1.00
Nigeria		45.1	45.0	1.00
Guinea		48.3	48.2	1.00
Ghana		31.1	31.7	0.98
Malawi		34.4	35.7	0.96
Liberia		36.3	40.4	0.90
**All countries**		**37.7**	**34.3**	**1.10**

**Table 6.** Selected determinants of neonatal mortality from multilevel logistic regression for 27 countries, 1990–2014.

Determinants	Odds ratio (95% CI) ^a^
Pregnancy characteristics	
Number of antenatal care (ANC) visits	
None	Ref
At least once	0.53 (0.50–0.56)***
Place of delivery	
Home	Ref
Health facility	0.99 (0.97–1.03)
Mother's age at birth	
< 20	Ref
20–24	0.65 (0.63–0.67)***
25–29	0.53 (0.51–0.55)***
30+	0.57 (0.55–0.60)***
Household characteristics	
Wealth quintile	
Lowest	Ref
Second	0.98 (0.95–1.01)
Third	1.01 (0.98–1.04)
Fourth	0.99 (0.96–1.03)
Highest	0.98 (0.94–1.03)
Residence	
Rural	Ref
Urban	0.86 (0.84–0.89)***
Woman characteristics	
Mother's education	
None	Ref
At least primary	0.81 (0.79–0.83)***
Married/Living together	
No	Ref
Yes	0.91 (0.88–0.95)***
Child-specific characteristics	
Birth order	
1	Ref
2–3	0.72 (0.70–0.74)***
4+	0.90 (0.87–0.94)***
Child's sex	
Male	Ref
Female	0.73 (0.71–0.74)***
Survey characteristics	
Round of survey period	
Earliest	Ref
Latest	0.73 (0.71–0.75)***
Round of surveys*ANC	
Survey period*No ANC visit	Ref
Survey period*At least one ANC visit	0.97 (0.90–1.04)

CI: confidence interval; Ref: Reference category; *** *p* < 0.001; ** *p* < 0.05; ^a^ Odds ratios were calculated using multivariate analysis with interaction between survey period and ANC attendance. A total of 1.1 million births were included in the analysis.

## 4. Discussion

The findings from this study suggest that NMR declined between the earliest and most recent surveys; from 46.0 deaths per 1000 live births during the earliest surveys to 33.4 deaths per 1000 live births. Although overall NMRs were 10% higher than expected, these findings have demonstrated the significant achievements made by countries since the 1990s in improving neonatal survival. That NMR was 2.2 times higher among births of women with no ANC than among births of women who had at least one ANC visit (42.5 vs 19.6 per 1000 live births) is consistent with small-scale studies that have documented the role of ANC interventions in reducing neonatal mortality (e.g. [3]). However, with an overall NMR of 37.7 (95% CI, 37.4–38.1) deaths per 1000 live births across all the 27 countries, the agenda to improve neonatal survival is not only continuing but also critical within the context of the Sustainable Development Goals.

The observed NMRs across countries show some important variations. For example, the lowest NMR among births of women with no ANC was in Namibia at 25.5 deaths per 1000 live births; and the highest in Mali at 63.9 deaths per 1000 live births. While this study does not account for all the unobserved factors that influence neonatal survival across countries, it is important to note that socio-cultural factors may play a role in the observed variations. Newborn care activities are different across societies and regions. Distance to facilities also varies across countries [6] so that even if women who did not attend ANC decide to take their newborns to a health facility for emergency care, the distance from their household to the facility may be shorter in some countries than in others. Even in countries where the distance is short, availability of qualified staff becomes a challenge. For example, the *World Health Statistics 2016 report* shows that the density of skilled health professionals per 10,000 population between 2005 and 2013 was 1.6 in Niger and 31.5 in Namibia [16]. Therefore, variations in the health workforce may explain some of the observed variations.

The burden of neonatal mortality among births of women with no ANC compared to births of women with at least one ANC ranged from a low of 1.4 in Namibia to 3.3 in Niger. This difference is substantial considering that across all countries, neonatal mortality of births among women with no ANC more than doubled compared to women with at least one ANC. This finding calls for continued efforts in ensuring that all women attend ANC irrespective of their socioeconomic background. About 37% of all the 27 countries had the ratio of NMR of births among women with no ANC double compared to births of women with at least one ANC. This finding was supported by regression results that showed that newborns whose mothers had at least one ANC visit were 47% less likely to die during the neonatal period than newborns whose mothers had no ANC. As countries start monitoring the health-related SDGs, it is important to commit to strategies such as the *Every Newborn* action plan that call for continued efforts to deploy interventions aimed at strengthening and investing in care during labour, birth and the first day and week of life as well as improving the quality of maternal and newborn care [2].

The finding that during the most recent surveys, the odds of newborns dying were 27% lower than during the earliest surveys is a positive development. Although progress to reduce neonatal mortality has been slow [9], a number of countries have achieved significant successes in reducing neonatal mortality. For example, between 1990 and 2015, Malawi registered a 55% reduction in NMR which was associated with, among other things, women's use of kangaroo mother care (KMC) in health facilities. KMC has helped improved neonatal survival in Malawi, a country where 40% of neonatal deaths result from complications from preterm birth. The number of institutions implementing KMC interventions in Malawi increased from 8 in 2008 to 121 by 2011 [17]. The effectiveness of KMC was associated with concomitant increases in facility births and other health system changes such as increased human resources and comprehensive deployment of high impact interventions for newborn survival [18]. Other countries such as Mali, Rwanda, and Uganda also reported the role of KMC in improving newborn survival [19] with a percentage decline in NMR between 1990 and 2015 of 48%, 54%, and 51%, respectively [9].

A notable program in improving newborn survival in sub-Saharan Africa is the Community-based Health Planning and Services (CHPS) which was launched by the Government of Ghana to scale-up a highly successful community health program that was developed by researchers at the Navrongo Health Research Centre in northern Ghana [20]. Follow-up strategies to strengthen the CHPS program such as the Ghana Essential Health Intervention Project (GEHIP) improved the CHPS model by expanding the range and quality of services for newborns; training community volunteers to conduct World Health Organization integrated management of childhood illness (IMCI); strengthening health information systems; enabling community nurses to manage and refer obstetric complications, among others. GEHIP interventions also included training volunteers in antibiotic therapy and malaria treatment to support communities that did not have access to IMCI services while waiting for scale up of the CHPS program at the national level. A number of studies using northern Ghana's Navrongo Demographic and Health Surveillance System (e.g. [21],[22]) showed that neonatal mortality had declined more gradually than post-neonatal mortality. This finding was attributed to interventions such as the CHPS model. GEHIP interventions were informed by prior interventions in Tanzania though the Tanzania Essential Health Interventions Project [23].

In 2000, the international community adopted the Millennium Development Goal to implement interventions and review policies to reduce under-5 mortality by two-thirds by 2015, from the 1990 levels. However, progress to achieve this goal was mixed: some countries had made drastic reductions while others had struggled to meet the deadlines. In particular, sub-Saharan Africa is a concern due to its huge burden of under-5 mortality. The decline in neonatal mortality over the 1990–2015 period had been slower than that of post-neonatal under-5 mortality (1–59 months): 47%, compared with 58% globally [9]. While progress to improve neonatal survival in a number of countries has been tremendous, efforts to encourage women to attend ANC remains critical. In addition to other interventions, ANC is one of the key interventions across the continuum of care to ensure that women receive appropriate advice and care for their newborns [24]. ANC enables women to receive care during pregnancy and those who do so are also likely to seek skilled care at birth and continue to seek early postnatal care.

The strengths and limitations of this study are worth noting. One of the main strengths of this study is the use of pooled large-scale population-based data from 27 countries in sub-Saharan Africa with diverse experiences. The availability of DHS from the early 1990s to 2014 offered an opportunity to examine the association between ANC and neonatal mortality. The DHS is also one of the key sources of data to monitor health-related indicators in sub-Saharan Africa. Nevertheless, limitations of the DHS include the lack of data on household income or expenditure, which would be one of the important input into the calculation of wealth index. The asset-based wealth index that was used as one of the control variable only provides a proxy indicator for the household SES [25]. Comparison of wealth index scores across countries is also problematic due to variability in the distribution of wealth and choice in the selection of assets to be included in the computation of the wealth index. Nevertheless, other studies in less developed countries have reported results that are robust than those using household-expenditure measures [26]. Despite this challenge, the wealth index is a preferred measure since it results from accumulation of income compared with the temporality and volatility associated with income and expenditure measures.

## 5. Conclusion

Irrespective of the country that was included in this analysis, women who attended ANC at least once had relatively lower odds of reporting neonatal death compared to women who did not attend ANC. Therefore, interventions should be tailored to ensure that women across all classes have a better understanding of the importance of ANC in improving maternal and child health. In addition, this awareness raising should be accompanied by interventions that eliminate barriers to access health care and ensure that no woman is left behind in receiving ANC.
